# *In Vitro* Effects of Combining Genistein with Aromatase Inhibitors: Concerns Regarding Its Consumption during Breast Cancer Treatment

**DOI:** 10.3390/molecules28134893

**Published:** 2023-06-21

**Authors:** Patrícia H. A. Bezerra, Cristina Amaral, Cristina F. Almeida, Georgina Correia-da-Silva, Maria Regina Torqueti, Natércia Teixeira

**Affiliations:** 1 Laboratory of Clinical Cytology, Department of Clinical Analyses, Toxicology and Food Science, School of Pharmaceutical Sciences of Ribeirão Preto, University of São Paulo, Ribeirão Preto 14040-903, SP, Brazil; paty.h.alves@alumni.usp.br; 2 UCIBIO.REQUIMTE, Laboratory of Biochemistry, Department of Biological Sciences, Faculty of Pharmacy, University of Porto, Rua Jorge Viterbo Ferreira, nº 228, 4050-313 Porto, Portugal; cristina-almeida96@hotmail.com (C.F.A.); george@ff.up.pt (G.C.-d.-S.); 3 Associate Laboratory i4HB—Institute for Health and Bioeconomy, Faculty of Pharmacy, University of Porto, Rua Jorge Viterbo Ferreira, nº 228, 4050-313 Porto, Portugal

**Keywords:** breast cancer, luminal A, genistein, Exemestane, Anastrozole, Letrozole, anticancer drugs, aromatase, estrogen receptor, androgen receptor

## Abstract

Introduction: The third-generation of aromatase inhibitors (AIs)—Exemestane (Exe), Letrozole (Let), and Anastrozole (Ana)—is the main therapeutic approach applied for estrogen receptor-positive (ER+) breast cancer (BC), the most common neoplasm in women worldwide. Despite their success, the development of resistance limits their efficacy. Genistein (G), a phytoestrogen present in soybean, has promising anticancer properties in ER+ BC cells, even when combined with anticancer drugs. Thus, the potential beneficial effects of combining G with AIs were investigated in sensitive (MCF7-aro) and resistant (LTEDaro) BC cells. Methods: The effects on cell proliferation and expression of aromatase, ERα/ERβ, and AR receptors were evaluated. Results: Unlike the combination of G with Ana or Let, which negatively affects the Ais’ therapeutic efficacy, G enhanced the anticancer properties of the steroidal AI Exe, increasing the antiproliferative effect and apoptosis relative to Exe. The hormone targets studied were not affected by this combination when compared with Exe. Conclusions: This is the first *in vitro* study that highlights the potential benefit of G as an adjuvant therapy with Exe, emphasizing, however, that soy derivatives widely used in the diet or applied as auxiliary medicines may increase the risk of adverse interactions with nonsteroidal AIs used in therapy.

## 1. Introduction

Luminal-type breast tumors are less aggressive and have a good prognosis, but they represent up to 70% of breast cancers (50% of Luminal A and 20% of Luminal B) in women worldwide [1]. For these cases, where estrogen (ER) and progesterone (PR) receptors are present, endocrine therapy is the best approach used in the clinic for both premenopausal and postmenopausal women [2]. Although Tamoxifen has been used as the first line of endocrine treatment since 1973 [3], clinical studies have shown that aromatase inhibitors (AIs) have higher clinical efficacy than tamoxifen, since they present prolonged disease-free survival and time to recurrence, as well as lower adverse side effects [4,5]. Thus, according to recent guidelines, the AIs are considered the first-line therapy for postmenopausal women, as well as for premenopausal women, after ovarian function suppression [6,7].

Aromatase (EC:1.14.14.14), which is encoded by the gene *CYP19A1*, is a key enzyme in the conversion of C19 androgens (testosterone and androstenedione) to C18 estrogens (estradiol and estrone) [8]. ER^+^ breast tumor tissues express higher levels of aromatase than non-tumor tissues [9], showing the importance of this enzyme for estrogen bioavailability in the tumor microenvironment. The third-generation AIs, currently used in the clinic, are Exemestane (Exe), a steroidal AI that irreversibly binds to aromatase, and Anastrozole (Ana) and Letrozole (Let), nonsteroidal AIs that bind reversibly to aromatase [4,5,10]. More specifically, Exe binds covalently to aromatase and degrades it through the proteasome, acting as a mechanism-based inhibitor, thus being considered a suicidal inhibitor [10,11]. In addition, this AI arrests the cell cycle progression of ER^+^ breast cancer cells and induces apoptosis and cytoprotective autophagy [12]. In ER^+^ breast cancer cells, Exe also causes overexpression of the androgen receptor (AR), which has an oncogenic role [13], and downregulates ERα, despite presenting a weak estrogen-like effect, through cooperation with AR [14]. On the other hand, Ana and Let are selective AIs, which contain a triazole ring that interacts noncovalently with the heme group of the CYP19A1 enzyme [8,15]. Furthermore, Let and Ana have been reported to cause antiproliferative effects through their action on the AR [16,17]. Recently, Augusto et al. showed that in addition to modulating AR and ERα levels without affecting their activation, they act on ER^+^ breast cancer cells as pure AIs [14]. Moreover, these nonsteroidal AIs induce cellular senescence on ER^+^ breast cancer cells, while Exe promotes cytoprotective autophagy, which impairs the induction of senescence [14]. In general, despite their good tolerability, when AIs are used for long periods, they are associated with severe adverse effects, such as bone and cardiovascular complications, as well as the development of resistance, which limits their therapeutic success [4,5,10]. The combination of AIs with mTOR and CDK4/6 inhibitors may improve cancer treatment by prolonging progression-free survival, though no expansion on overall survival is observed, and resistance still occurs [7,18,19,20]. Therefore, the search for new molecules that can improve the efficacy of conventional therapy is ongoing.

Genistein (G), an isoflavone of soybeans, has shown antitumor activity *in vitro* and *in vivo* for various types of carcinoma, such as metastatic bladder, cervical, prostate, kidney, and breast cancer [21,22,23]. This nonsteroidal phytoestrogen is structurally similar to 17β-estradiol, presenting great affinity for ERs, preferably for ERβ, which is related to the inhibition of cell proliferation. Although it has been reported that this isoflavone can inhibit several enzymes involved in estrogen biosynthesis, including aromatase, its effects have been controversial [24,25,26,27,28,29]. It has demonstrated its inhibitory activity on tyrosine kinase enzymes and DNA topoisomerase II, which are related to cell proliferation, as well as on stimulatory sex hormone-bound globulin (SHBG), associated with decreased circulating active estrogen [22,30]. In addition, in the MCF-7 cell line, it was shown that biotransformed soybeans, with a higher amount of G and Daidzein (D), induced apoptosis through a decrease in Bcl-2/Bad and Bcl-xl/Bad and an increase in proapoptotic proteins of the intrinsic pathway [31]. Furthermore, in MCF-7aro cells, our group also reported that G impaired cancer cell growth, although it was considered a weak AI [29].

Regarding the pharmacological combination of G with conventional therapy, several studies indicate a synergic effect of this phytoestrogen with some chemotherapeutics, such as Doxorubicin and Docetaxel in both estrogen-dependent and estrogen-independent breast tumor cell models [21]. However, the literature only shows conclusive data on endocrine therapies for Tamoxifen, where *in vivo* assays indicate that G increases the response of breast tumors to Tamoxifen when consumed via diet from birth [32]. Nevertheless, besides suggesting that soy-based G-enriched supplements can affect the efficacy of AIs on breast cancer cells [33], this study only investigated the negative interaction with the nonsteroidal AI Fadrozole. Thus, the real impact of the dietary consumption of this type of phytoestrogens, such as G, with the AIs applied in the clinic is yet to be understood. Therefore, this study evaluated the impact of combining G with the AIs used in the clinic, such as Ana, Let, and Exe, assessing whether their combination could improve the effectiveness of these AIs and, consequently, the treatment of breast cancer, allowing for a new perspective of the clinical application of this phytoestrogen.

## 2. Results

### 2.1. Genistein Decreases Cell Viability of ER+ Breast Cancer Cells

**Figure 1:**
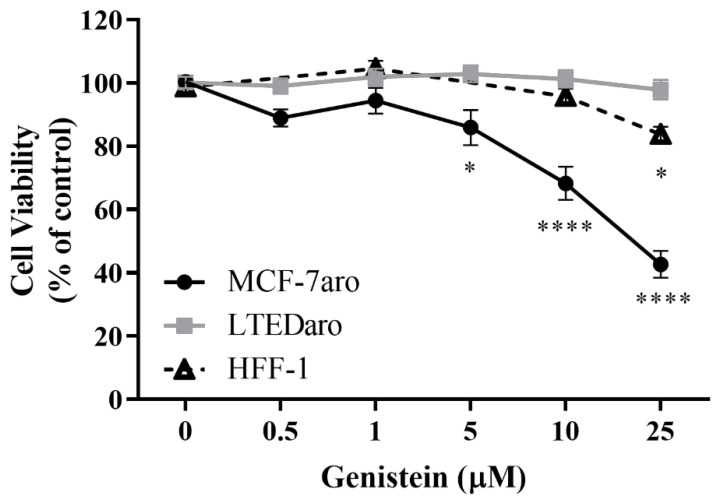
Effects of Genistein (G) on MCF-7aro, LTEDaro, and HFF-1 cells. Cells were treated with different concentrations of G (0.5–25 µM) for 3 days. In MCF-7aro cells, testosterone (T) (1 nM) was also applied, which was considered the control (100% of cell viability), while for the other cell lines, cells without G treatment were considered the control group (100% of cell viability). Data are presented as mean ± SEM of at least three independent experiments, performed in triplicate. Significant differences between control and treated cells are designated as * (*p* < 0.05), **** (*p* < 0.0001).

The effect of Genistein (G) on the viability of the non-tumor HFF-1 cells and on the sensitive and resistant ER^+^ breast cancer cell lines, MCF-7aro and LTEDaro was evaluated by MTT assay after 3 days of treatment. The results (Figure 1) showed that G was able to significantly decrease (*p* < 0.05; *p* < 0.0001) the viability of MCF-7aro cells in a dose-dependent manner. However, no significant anticancer effect was observed on the LTEDaro cells. Moreover, as cytotoxicity in HFF-1 cells was observed for the highest concentration studied (25 µM; *p* < 0.05), this dose was not used for the subsequent experiments.

### 2.2. Genistein Potentiates Exemestane Anticancer Effect

**Figure 2:**
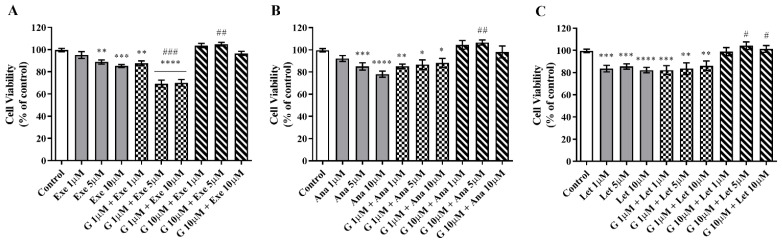
Effects of the combination of Genistein (G) with AIs Exemestane (Exe) (**A**), Anastrozole (Ana) (**B**), and Letrozole (Let) (**C**) on the viability of MCF-7aro cells. Cells stimulated with testosterone (T; 1 nM) were treated with G (1 or 10 µM) and Exe (1, 5 or 10 µM), Ana (1, 5 or 10 µM), or Let (1, 5 or 10 µM) for 3 days. Cells only treated with T were considered the control group (100% of cell viability). Data are presented as mean ± SEM of at least three independent experiments, performed in triplicate. Significant differences between control and treated cells are designated as * (*p* < 0.05), ** (*p* < 0.01), *** (*p* < 0.001), and **** (*p* < 0.0001), while differences between the AIs and the combination of G with AIs are indicated as # (*p* < 0.05), ## (*p* < 0.01) and ### (*p* < 0.001).

The impact of G on the cell viability induced by the three AIs used in the clinic was studied using different combinations of G and AIs. The combination of G at 1 µM significantly potentiated (*p* < 0.001) the anticancer effect of Exe at 5 and 10 µM (Figure 2A). However, this was not observed for the combination of Exe with the higher dose of G (10 µM), which even reversed the effect of the AI alone. The latter effect was also observed for the nonsteroidal AIs, Ana and Let (Figure 2B,C). In fact, contrary to Exe, even for the lower concentration of G, no beneficial effect was observed for the nonsteroidal AIs. Thus, given these results, subsequent experiments were only conducted for G at 1 µM associated with Exe.

In order to highlight the mechanism behind the combination of G with Exe, the cell cycle progression was evaluated by flow cytometry. Table 1 shows that G alone at 1 µM caused a significant (*p* < 0.05) G_2_/M cell cycle arrest, while significantly decreasing (*p* < 0.0001) the S phase in a dose-dependent manner Treatment with Exe revealed a significant (*p* < 0.0001) increase in the G_0_/G_1_ phase while decreasing both the S (*p* < 0.0001) and G_2_/M (*p* < 0.001) phases. This effect was potentiated when combined with G because significant (*p* < 0.05) differences between Exe with or without G in the S phase were observed.

**Figure 3:**
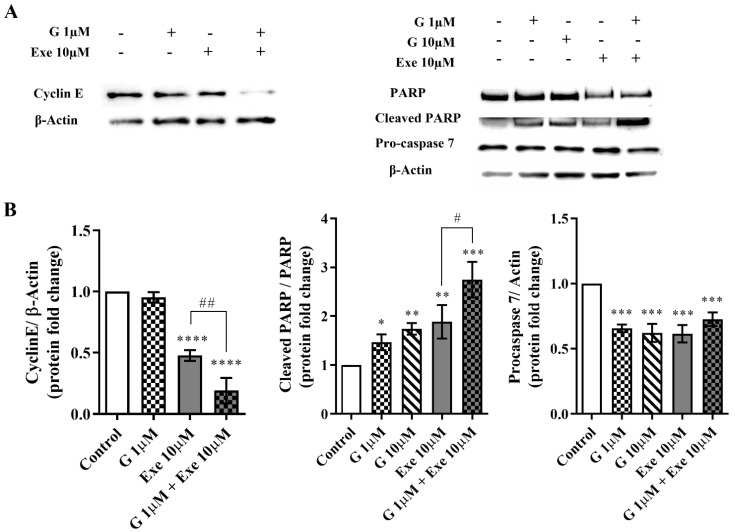
Effects of the combination of Genistein (G) with Exemestane (Exe) on the expression levels of cyclin E, cleaved PARP, and pro-caspase-7 in MCF-7aro cells. Cells were stimulated with Testosterone (T) (1 nM) and treated with G (1 or 10 µM) with or without Exe (10 µM) for 3 days. Cells treated only with T were considered the control group. Representative Western blot (**A**) and densitometric analysis (**B**) of cyclin E, cleaved PARP, and pro-caspase-7. β-Actin or total PARP was used as a loading control. The protein expression obtained for treated cells was normalized in relation to the protein expression of the control. The results are presented as mean ± SEM of at least three independent experiments. Significant differences between control and treated cells are designated as * (*p* < 0.05), ** (*p* < 0.01), *** (*p* < 0.001) and **** (*p* < 0.0001), while differences between Exe and its combination with G are indicated as # (*p* < 0.05) and ## (*p* < 0.01).

In order to understand the antiproliferative effect, the expression of cyclin E was investigated by Western blot. Exe *per se* significantly (*p* < 0.0001) decreased the expression levels of cyclin E, which is in accordance with our previous research [14]. More importantly, the combination of Exe with G extensively reduced (*p* < 0.0001) cyclin E levels when compared with Exe (*p* < 0.01) or G alone (Figure 3A). In addition, as a reduction in MCF-7aro cell viability can also be associated with cell death, the expression levels of PARP and pro-caspase-7, two biomarkers of apoptotic cell death, were also explored. As demonstrated in Figure 3, G *per se* caused a significant (*p* < 0.05 and *p* < 0.01) increase in the levels of cleaved PARP and a significant (*p* < 0.001) reduction in pro-caspase-7. In agreement with our previous studies [12,13,14], Exe also significantly increased (*p* < 0.01) the expression levels of cleaved PARP and decreased (*p* < 0.001) the levels of pro-caspase-7. In relation to the combination, although no significant differences in pro-caspase-7 were noted when compared with the AI or G alone, the combination promoted a significant (*p* < 0.001) increase in the ratio of cleaved PARP/PARP, with differences being observed between the combination and Exe-treated cells (*p* < 0.05) (Figure 3).

### 2.3. Genistein Affects Aromatase and Hormone Receptors’ Expressions

In order to elucidate the involvement of the main targets of ER^+^ breast cancer, aromatase and hormone receptors (ERα, ERβ, and AR) and cells treated with Exe (10 µM) with or without G (1–10 µM) were also incubated with the aromatase product (estrogen, E_2_) or stimulated with T and also incubated with the antagonists of ERα (ICI), ERβ (PHTPP), and AR (CDX). In addition, the expression levels of the enzyme aromatase and the receptors were evaluated by Western blot. 

**Figure 4:**
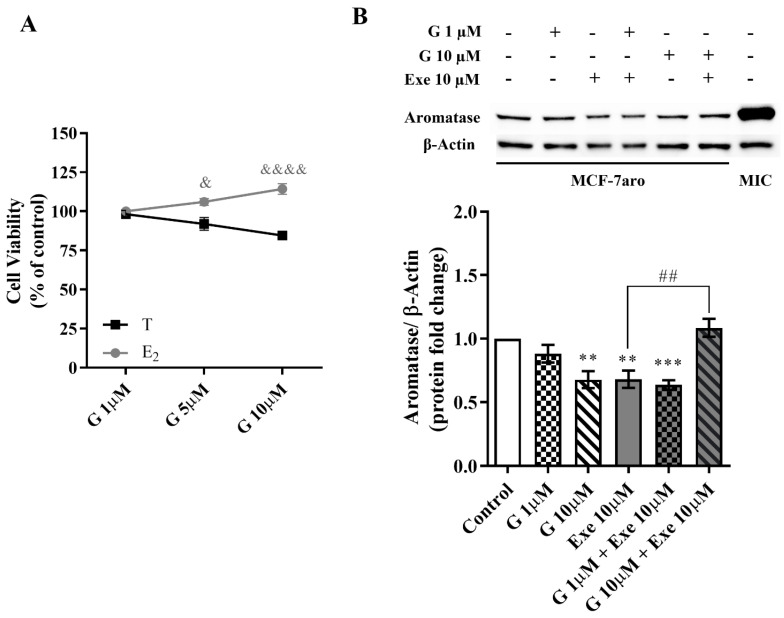
Influence of aromatase on the effects induced by Genistein (G) alone and when combined with Exemestane (Exe) in MCF-7aro cells. (**A**) Effects of G (1–10 µM) on the viability of MCF-7aro cells stimulated with Testosterone (T) (1 nM) or estradiol (E_2_) (1 nM), after 3 days. Cells treated only with T or E_2_ were considered the control group. (**B**) Representative Western blot and densitometric analysis of the effects of G (1 and 10 µM) with or without Exe (10 µM) on the expression levels of aromatase, after 8 h exposure. Β-Actin was used as a loading control. The protein expression obtained for treated cells was normalized in relation to the protein expression of the control (T-treated cells). The results are presented as mean ± SEM of at least three independent experiments. Significant differences between control and treated cells are designated as ** (*p* < 0.01) and *** (*p* < 0.001) while differences between cells treated with G and stimulated with T versus E_2_ are denoted as & (*p* < 0.05) and &&&& (*p* < 0.0001), and differences between Exe and its combination with G are indicated as ## (*p* < 0.01). MIC: placenta microsomes (positive control).

In relation to aromatase, results showed that in the presence of E_2_, G significantly lost the ability to decrease MCF-7aro cell viability (Figure 4A) when compared with cells stimulated with T, which suggests an effect on cells that is aromatase-dependent. In accordance with previous studies [11,14,34,35,36,37,38], Exe decreases the aromatase levels in MCF-7aro cells. Similarly to Exe, G at 10 µM was also able to significantly (*p* < 0.001) reduce the expression levels of aromatase (Figure 4B). In relation to the combination of G with Exe, the combination with G at 1 µM presented a similar behavior as Exe, i.e., a reduction in aromatase levels (*p* < 0.001), while G at 10 µM significantly (*p* < 0.01) reversed the effect induced by Exe on aromatase levels, thus displaying the behavior similar to control in the latter case (Figure 4B).

**Figure 5:**
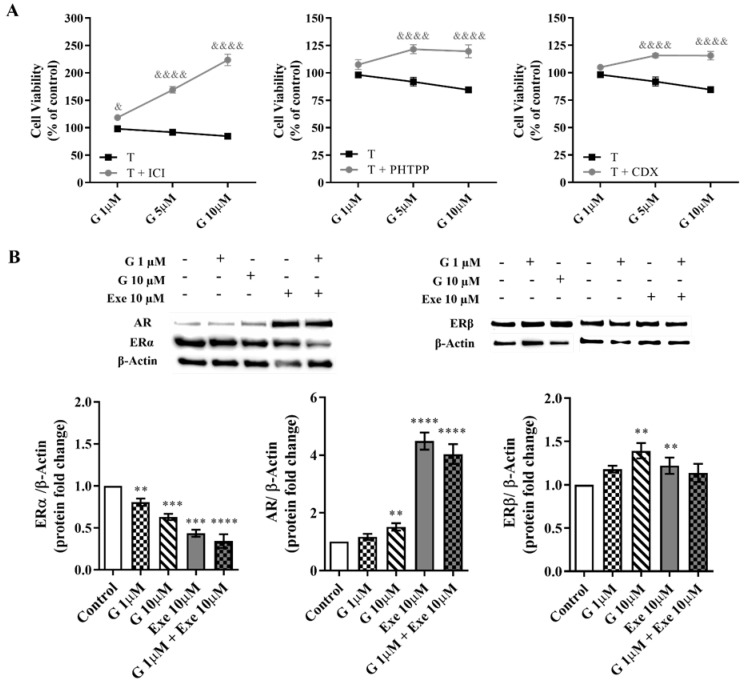
Influence of hormone receptors ERα, ERβ, and AR on the effects induced by Genistein (G) alone and when combined with Exemestane (Exe) in MCF-7aro cells. (**A**) Effects of G (1–10 µM) on the viability of MCF-7aro cells stimulated with Testosterone (T) (1 nM) and treated with or without ICI (100 nM) or PHTPP (1 µM) or CDX (1 µM), after 3 days. Cells treated only with T with or without ICI, PHTPP, or CDX were considered the control group. (**B**) Representative Western blot and densitometric analysis of the effects of G (1 and 10 µM) with or without Exe (10 µM) on the expression levels of ERα, ERβ, and AR, after 3 days. β-Actin was used as a loading control. The protein expression obtained for treated cells was normalized in relation to the protein expression of the control (T-treated cells). The results are presented as mean ± SEM of at least three inde-pendent experiments. Significant differences between control and treated cells are designated as ** (*p* < 0.01), *** (*p* < 0.001), and **** (*p* < 0.0001), while differences between cells treated with G and incubated with T with or without ICI, PHTPP or CDX are denoted as & (*p* < 0.05) and &&&& (*p* < 0.0001).

Regarding the effect of G on hormone receptors, the antagonists of ERα (ICI 182,780), ERβ (PHTPP), or AR (CDX) significantly (*p* < 0.0001) prevented the ability of G at higher doses (5–10 µM) to decrease the viability of MCF-7aro cells (Figure 5A); these effects suggest an ERrα-/ERβ- and AR-dependent mechanism of action of G on these cells. The protein analysis of ERα revealed that G at 1 µM and 10 µM is able to significantly (*p* < 0.01, *p* < 0.0001) decrease ERα expression. However, only at the highest dose, G increases the expression levels of both ERβ (*p* < 0.01) and AR (*p* < 0.01) (Figure 5B). In accordance with previous studies by our group, Exe also decreases the expression levels of ERα [14] and increases AR levels [13]. Curiously, it can also increase the expression levels of ERβ. However, no significant difference in the expression of all hormone receptors was noted when G was combined with Exe or in relation to Exe alone—the behavior being, in fact, similar to Exe (Figure 5B).

## 3. Methods

### 3.1. Cell Culture

As a non-tumor cell line, a human foreskin fibroblast cell line (HFF-1) was also used, cultured in Eagle’s minimum essential medium (MEM) without phenol red, supplemented with 1 mmol/L sodium pyruvate, 2 mmol/L of L-glutamine, 1% penicillin-streptomycin-amphotericin B, and 10% of fetal bovine serum (FBS).

MCF-7aro cells are an ER^+^ aromatase-overexpressing human breast cancer cell line obtained from the parental human epithelial ER^+^ breast cancer cell line (MCF-7 cells) after stable transfection with the human placental aromatase gene and using the Geneticin (G418) selection, as previously described [39,40]. These cells, which are representative of the luminal A subtype, were cultured in Eagle’s minimum essential medium (MEM) (Gibco Invitrogen Co., Paisley, Scotland, UK) supplemented with 1 mmol/L sodium pyruvate, 1% penicillin-streptomycin-amphotericin B, 100 μg/mL G418, and 10% heat-inactivated FBS (Gibco Invitrogen Co., Paisley, Scotland, UK). To avoid the interference of the estrogen-like effects of phenol red and the hormones present in FBS, 3 days before the experiments, these cells were cultured in an estrogen-free MEM without phenol red (Gibco Invitrogen Co., Paisley, Scotland, UK), containing 5% of heat-inactivated charcoal pretreated fetal bovine serum (CFBS), 1 mmol/L of sodium pyruvate, 1% of penicillin-streptomycin-amphotericin B and 2 mmol/L of L-glutamine, as previously reported [13]. In addition, for the experiments, these cells were also stimulated with testosterone (T) or with estradiol (E2) (Sigma-Aldrich Co., Saint Louis, MO, USA), the aromatase substrate, or the aromatase product at 1 nM, respectively, which were used as proliferation-inducing agents.

To mimic the late stage of acquired resistance to the AIs used in the clinic, LTEDaro cells, along-term estrogen-deprived human ER^+^ breast cancer cell line, were used [41,42]. These cells were cultured in Eagle’s minimum essential medium (MEM) without phenol red and supplemented with Earle’s salts and with 1 mmol/L sodium pyruvate, 1% penicillin–streptomycin-amphotericin B, 1% L-Glutamine, 100 μg/ml G418, and 10% of CFBS, as previously reported [13]. Both cell lines were kindly provided by Professor Shiuan Chen (Beckman Research Institute, City of Hope, Duarte, CA, USA). 

All the cell lines applied in this study were maintained at 37 °C and 5% CO_2_ atmosphere.

Stock solutions of testosterone (T) and estradiol (E_2_) were prepared in absolute ethanol (Sigma-Aldrich Co., Saint Louis, MO, USA). Exemestane (Exe) (Carbosynth, Berkshire, UK), Genistein (G) from *Glycine max*, Anastrazole (Ana), Letrozole (Let), Casodex (CDX), ICI 182,780 and 4-[2-phenyl-5,7, bis (trifluoromethyl) pyrazole [1,5-a]pyrimidin-3-yl]phenol (PHTPP) (Sigma-Aldrich Co., Saint Louis, MO, USA) were prepared in 100% DMSO (Sigma-Aldrich Co., Saint Louis, MO, USA). All these solutions were stored at −20 °C, and fresh samples were prepared before each experiment. Final concentrations of ethanol and DMSO in the culture medium were lower than 0.05% and 0.01%, respectively. All the controls contained these vehicles in these culture conditions.

### 3.2. Cell Viability Assay

Cell viability was determined using the MTT assay. Cells were seeded into 96-well culture plates (2 × 10^4^ cells/well) and treated with G (0.5–25 µM), with or without Exe, Ana, or Let (1, 5, and 10 µM) for 3 days. It should be pointed out that MCF-7aro cells were also stimulated with T (1 nM), as described above. In addition, to elucidate possible mechanisms of action of G, MCF-7aro cells were also treated with Estradiol (E_2_) (1 nM) or with the selective estrogen receptor alpha downregulator ICI 182,780 (100 nM), or with the estrogen receptor beta inhibitor PHTPP (1 µM) or with the androgen receptor antagonist CDX (1 µM), co-administered with G. After the incubation time of each experiment, cells were incubated with MTT (0.5 mg/mL) (Sigma-Aldrich Co., Saint Louis, MO, USA) and quantified spectrophotometrically in a Biotek Synergy HTX Multi-Mode Microplate Reader (Biotek Instruments, Winooski, VT, USA). Cells without G treatment were considered the control group.

### 3.3. Cell Cycle Progression Assay

To study the effects of G on MCF-7aro cell cycle progression, the DNA content was assessed through labeling with propidium iodide (PI) and by performing flow cytometry. Cells (7 × 10^5^ cells/mL) stimulated with T (1 nM) were incubated with G (1 µM), Exe (10 µM), or with G plus Exe, for 3 days. Cells only treated with T were considered the control group. Following treatments, cells were fixed with cold 70% EtOH and stained with a DNA labeling solution (Propidium Iodide at 5 μg/mL, 0.1% Triton X-100 and 200 μg/mL DNase-free RNAse A) (Sigma–Aldrich Co., Saint Louis, MO, USA), as previously described [39]. DNA content was analyzed by flow cytometry based on the acquisition of 40,000 events in a BD Accuri C6 cytometer (San Jose, CA, USA). Detectors for the three fluorescence channels (FL-1, FL-2, and FL-3) and for forward (FSC) and side (SSC) light scatter were set on a linear scale. Debris, cell doublets, and aggregates were gated out using a two-parameter plot of FL-2-Area to FL-2-Width of PI fluorescence. Data were analyzed using the BD Accuri™ C6 analysis software. The antiproliferative effects were indicated by the percentage of cells in the G_0_/G_1_, S, and G_2_/M phases of the cell cycle.

### 3.4. Protein Expression

The expression levels of cyclin E, PARP, pro-caspase-7, aromatase, estrogen receptor α (ERα), estrogen receptor β (ERβ), and androgen receptor (AR) were evaluated by Western blot. For this, MCF-7aro cells (7 × 10^5^ cells/ml) were cultured in 6-well plates and treated with G (1 μM) with or without Exe (10 µM) for 3 days. Cells without treatment were designated as control. Following treatments, cells’ lysates were analyzed by Western blot. An amount of 50 μg of protein sample was subjected to 10% of SDS-PAGE and then transferred to nitrocellulose membranes. For the immunodetection, the mouse monoclonals antibodies anti-pro-caspase-7 (1:200, sc-56063), anti-cyclin E (1:200, sc-377100), anti-cleaved-PARP (1:200, sc-56196), anti-CYP19A1 (1:200, sc-374176), anti-ERα (1:200, sc-8002), anti-AR (1:200, sc-7305) (Santa Cruz Biotechnology, Santa Cruz, CA, USA), and anti-ERβ (1:200, PPZ0506) (Thermo Fisher, Waltham, MA, USA) were used as primary antibodies, whereas the peroxidase-conjugated goat anti-mouse (1:2000, G21040) (Thermo Fisher, Waltham, MA, USA) was used as secondary antibodies. To control loading variations, a mouse monoclonal anti-β-actin antibody (1:500, sc-47778) (Santa Cruz Biotechnology, Santa Cruz, CA, USA) was applied. Immunoreactive bands were detected using a chemiluminescent substrate Super Signal West Pico (Pierce, Rockford, IL, USA) and a ChemiDoc™ Touch Imaging System (Bio-Rad Laboratories, Melville, NY, USA). 

### 3.5. Statistical Analysis

Assays were carried out in triplicate in at least three independent experiments, and all the data were expressed as the mean ± SEM. Statistical analysis was performed with GraphPad Prism 7^®^ software through analysis of variance (ANOVA) followed by Dunnet and Bonferroni post hoc tests for multiple comparisons. Values of *p* < 0.05 were considered statistically significant.

## 4. Discussion

Several *in vitro* and *in vivo* studies have highlighted the anticancer properties of Genistein (G) in different types of cancer, including breast cancer, by different mechanisms [21,22,23]. However, the mechanism of action of G in breast cancer is still controversial. In addition, G becomes an interesting molecule when combined with chemotherapeutic agents by increasing the antitumor efficacy while decreasing treatment toxicity [43]. This isoflavone has already demonstrated a synergistic association with Doxorubicin, increasing its effects against MCF-7/ADR cells, a Luminal multidrug-resistant breast cancer cell model, through cell cycle retention and induction of apoptosis [44]. Further, G acts synergistically with the SERM Ormeloxifene in MCF-7 and MDA-MB-231 cell lines, among other breast tumor cells [45]. A similar effect is also observed in association with eicosapentaenoic acid, a component of fish oil that is being studied for its anticancer activity [46]. Regarding the combination with Tamoxifen, G at low concentrations (1–10 µg/mL) has an additive inhibitory effect on breast cancer cells MCF-7, MDA-MB-231, and MDA-MB-435 [47]. Amaral C. et al. previously demonstrated that soybean extracts inhibit the growth of ER^+^ breast cancer cells, decreasing MCF-7aro cell viability and DNA synthesis, as well as affecting cell cycle progression, making G the isoflavone responsible for the effects induced by the soy extract on these cells [29]. Therefore, considering our previous work and the potential of G either alone or when combined with anticancer drugs, the present study investigated the ability of G to improve the anticancer properties of the AIs used in the clinic (Exemestane (Exe), Anastrozole (Ana), and Letrozole (Let)), in sensitive ER^+^ breast cancer cells, as well as its ability to re-sensitize resistant breast cancer cells. Furthermore, with this study, we also intend to provide relevant information on the mechanism of action of G in ER^+^ breast cancer cells. In fact, our data demonstrate that only in the MCF-7aro cells, G caused anti-growth effects, not affecting the viability of the resistant or non-tumoral cells. This suggests that G only presents anticancer potential in sensitive ER+ breast cancer cells, while not acting as a promising molecule in a resistant setting. Therefore, the potential benefits of combining G with the AIs Exe, Ana, and Let were only explored in the sensitive MCF-7aro cell line. Our results suggest that G at low doses may improve the efficacy of the steroidal AI Exe, but not of Ana or Let. In accordance with our results, a xenographic model of estrogen-dependent MCF-7Ca cells that also overexpress aromatase showed that in the presence of dietary concentrations of G, the effects of Let were inhibited [48]. In addition, G can also negatively affect the efficacy of the nonsteroidal AI fadrozole on breast cancer cells [33]. A possible explanation for the difference observed in the effects of AIs upon co-treatment with G can be the chemical nature of the AIs and the potential competition of this isoflavone with the nonsteroidal compounds at the aromatase binding site, preventing its anti-aromatase activities. We have previously demonstrated that G decreases aromatase activity in human placental microsomes [29]. In addition, this study showed for the first time that G presents an aromatase-dependent effect on ER+ breast cancer cells, since G, at 10 µM, has the ability to decrease the expression levels of aromatase in MCF-7aro cells, an effect similar to Exe [11,14,34,35,36,37,38] and opposite to those already reported for Ana and Let [14]. Interestingly, only the combination with G at 10 µM prevented the degradation of aromatase detected for both G and Exe alone, which emphasizes that the loss of clinical benefit for the highest dose of G may be related to the lack of effect on aromatase. Despite that, for the combination with G at low doses, a decrease in aromatase levels continues to persist in a similar way to Exe.

In contrast, the combination of G with Exe further increases the antiproliferative effect of Exe by decreasing the number of cells in the S phase but also causing an increment in the G_0_/G_1_ phase. The decrease in cyclin E expression with the combined treatment indicates the inability to transition between the G_0_/G_1_ and S phases since such progression is finely controlled by a high expression of this protein [49]. Previous studies have shown that high levels of cyclin E indicate a worse prognosis for patients with breast cancer, leading to a shorter 5-year survival [49], and its decrease is a desirable effect of the antitumor therapy, a fact achieved by this combination. In addition, G alone arrested MCF-7aro cell cycle progression at the G_2_/M phase. In accordance with our study, Li Z. et al. also reported that G arrested cell cycle at the G_2_/M phase of the breast cancer cells MDA-MB-231 [50]. Moreover, results show that G alone increases the expression of cleaved-PARP and decreases the expression of pro-caspase-7, both hallmarks of apoptosis, which indicates that G induces apoptosis of MCF-7aro cells. Other groups have also reported the induction of apoptosis of breast cancer cells by this isoflavone [22,23,51,52,53,54,55]. More importantly, in combination with Exe, G significantly increases the expression of cleaved-PARP, in relation to Exe. Thus, these data suggest that the combination of Exe with 1 µM of G increases the levels of apoptosis induced by Exe, as well as its antiproliferative effects, both beneficial anticancer effects to improve Exe treatment. Studies have shown that in front of a specific stimulus, such as DNA damage, hypoxia, or treatment with certain chemotherapeutics, the p53 gene, a tumor suppressor, may be activated, leading to the overexpression of the p21 gene and consequent retention in the cell cycle, by the inhibition of cyclins, resulting in a non-transition between the G1 and S phases. Finally, upon p53 activation, there is inhibition of the retinoblastoma protein (pRb), which results in proapoptotic signaling [56]. Indeed, there is evidence that G induces p53 activation, which, consequently, leads to cancer cell death [57,58,59,60].

On the other hand, it has been described that G is able to modulate the hormone receptors, ERα, ERβ, and AR [61]. Although studies showed that G, compared with estradiol, binds with 87% affinity to ERβ and only with 4% to ERα [62], the results of this study demonstrate that both ERs may contribute to the anticancer activity of this isoflavone in MCF-7aro cells. In fact, downregulation of ERα and overexpression of ERβ were observed in MCF-7aro cells after G treatment, which indicates a promising anticarcinogenic effect as ERα is associated with proliferation whereas ERβ is related to cell death [63,64]. This isoflavone also increased the AR expression in MCF-7aro cells, which may be due to hormonal compensation for the suppression of ERα [13,64]. In addition, the ERα, ERβ, and AR antagonists were able to reverse the effects of G on MCF-7aro cell viability, suggesting that G has antiproliferative ERα-/ERβ-and AR-dependent effects. However, with the combination of G with Exe, the expression of the hormone receptors was similar to those induced by Exe *per se*, suggesting that the mechanism underlying the increased antiproliferative effects of the combination may not be related to the hormone receptors.

This study highlights the anticancer potential of G as a multi-target compound in ER+ breast cancer cells, of the luminal subtype A, as this isoflavone interrupts the cell cycle, induces apoptosis, exerting antiproliferative effects by targeting aromatase, ERα, ERβ, and AR, which from a clinical point of view is a therapeutic advantage [4]. The duplicity of G as a therapeutic agent or endocrine disruptor has recently been reported [65]. In addition, this is the first study exploring the benefits of combining G with the AIs currently applied in the clinic. Curiously, the results highlight only a beneficial anticancer effect as adjuvant therapy for low doses of G only when associated with the steroidal AI Exe. However, when used in combination with nonsteroidal AIs, independent of the dose, G can negatively affect their therapeutic efficacy. These observations are extremely important from a clinical perspective, as they suggest that soybeans or soy-based products, widely consumed in the diet, as well as G used as an auxiliary medicinal for women’s diseases [65], may not be consumed whenever nonsteroidal AIs are used in therapy. Nevertheless, further studies must be performed to clarify this, as this is only an *in vitro* study based on a specific cell type model. Moreover, it should be pointed out that G is converted into genistein glucuronide and sulphate in the intestine [23], and it is known that isoflavone glucuronides might compete with endogenous estrogen to inhibit estrogen-dependent proliferation of cancer cells [66]. Thus, further studies should be performed in order to elucidate this issue.

## Figures and Tables

**Table 1:** Effects on cell cycle of MCF-7aro of Genistein (G) alone or in combination with Exemestane (Exe) for 3 days.

Treatment	G_0_/G_1_ (%)	S (%)	G_2_/M (%)
Control	76.71 ± 0.42	8.26 ± 0.23	14.94 ± 0.36
Gen 1 µM	78.82 ± 0.41	3.54 ± 0.11 ****	17.01 ± 0.39 *
Exe 10 µM	85.79 ± 0.29 ****	2.61 ± 0.23 ****	11.18 ± 0.42 ***
Gen 1 µM + Exe 10 µM	88.00 ± 0.39	1.40 ± 0.13 #	10.37 ± 0.28

Data represent mean ± SEM of three independent experiments. Significant differences between control and treated cells are denoted as * (*p* < 0.05), *** (*p* < 0.01), and **** (*p* < 0.001), while differences between Exe and its combination with G are indicated as # (*p* < 0.05).

## Data Availability

Not applicable.
